# Perceptual factors contribute more than acoustical factors to sound localization abilities with virtual sources

**DOI:** 10.3389/fnins.2014.00451

**Published:** 2015-01-29

**Authors:** Guillaume Andéol, Sophie Savel, Anne Guillaume

**Affiliations:** ^1^Département Action et Cognition en Situation Opérationnelle, Institut de Recherche Biomédicale des ArméesBrétigny sur Orge, France; ^2^Laboratoire de Mécanique et d'Acoustique, Centre National de la Recherche Scientifique, UPR 7051, Equipe Sons, Aix-Marseille Université, Centrale MarseilleMarseille, France; ^3^Laboratoire d'Accidentologie, de Biomécanique et d'Étude du Comportement HumainNanterre, France

**Keywords:** sound localization, perceptual learning, procedural learning, head-related transfer function, individual differences

## Abstract

Human sound localization abilities rely on binaural and spectral cues. Spectral cues arise from interactions between the sound wave and the listener's body (head-related transfer function, HRTF). Large individual differences were reported in localization abilities, even in young normal-hearing adults. Several studies have attempted to determine whether localization abilities depend mostly on acoustical cues or on perceptual processes involved in the analysis of these cues. These studies have yielded inconsistent findings, which could result from methodological issues. In this study, we measured sound localization performance with normal and modified acoustical cues (i.e., with individual and non-individual HRTFs, respectively) in 20 naïve listeners. Test conditions were chosen to address most methodological issues from past studies. Procedural training was provided prior to sound localization tests. The results showed no direct relationship between behavioral results and an acoustical metrics (spectral-shape prominence of individual HRTFs). Despite uncertainties due to technical issues with the normalization of the HRTFs, large acoustical differences between individual and non-individual HRTFs appeared to be needed to produce behavioral effects. A subset of 15 listeners then trained in the sound localization task with individual HRTFs. Training included either visual correct-answer feedback (for the test group) or no feedback (for the control group), and was assumed to elicit perceptual learning for the test group only. Few listeners from the control group, but most listeners from the test group, showed significant training-induced learning. For the test group, learning was related to pre-training performance (i.e., the poorer the pre-training performance, the greater the learning amount) and was retained after 1 month. The results are interpreted as being in favor of a larger contribution of perceptual factors than of acoustical factors to sound localization abilities with virtual sources.

## Introduction

Individuals receive information about their environment mainly via the visual and auditory sensory modalities. The auditory system has lower spatial resolution than the visual system, but allows perception beyond the visual field and in darkness. However, there is no direct encoding of space in the auditory system. Auditory space perception relies on the processing of binaural cues (i.e., interaural differences in the level and time of arrival of the incoming sound wave) for the left/right dimension, and spectral cues (i.e., filtering of the incoming sound wave by the listener's upper body, which corresponds to the head-related transfer function, HRTF) for the up/down and front/back dimensions. These direction-dependent cues are transformed into a complex audio-spatial map, which depends on anatomical characteristics and develops through experience with sensory—mainly visual (King, 2009)—feedback. Audio-spatial maps have been found to be highly plastic throughout life (Clifton et al., 1988; Hofman et al., 1998; Otte et al., 2013). Experience-dependent plasticity provides a potential neural basis for training-induced perceptual improvements in performance.

Large individual differences in localization ability have been reported, even in young normal-hearing adults (Wightman and Kistler, 1989; Makous and Middlebrooks, 1990; Wenzel et al., 1993; Populin, 2008; Savel, 2009). These individual differences were mainly observed under experimental conditions that are assumed to involve spectral cues: localization in the up/down and front/back dimensions (Wightman and Kistler, 1989; Wenzel et al., 1993) and in noise (Best et al., 2005). Two main contributing factors to localization abilities have therefore been proposed: spectral cues, and perceptual processes involved in the analysis of these cues. Several studies have assessed the contributions of these two factors separately.

It has been proposed that localization abilities depend mainly on the physical saliency of the acoustical cues carried by HRTFs. According to this hypothesis, the performance of listeners with poorer abilities would be hampered by insufficiently salient spectral cues. This hypothesis was initially supported by the finding that listeners with poor localization performance substantially improved when these listeners used the HRTFs of other listeners who had better performance (Butler and Belendiuk, 1977; Wenzel et al., 1988; Asano et al., 1990). However, the physical saliency of spectral cues was not quantified, and more recent studies, involving more listeners, did not confirm this finding (Møller et al., 1996; Middlebrooks, 1999b). A recent study assessed the spectral shape prominence of 15 individual HRTFs, and found no relationship between this acoustical metrics and localization performance in noise (Andéol et al., 2013).

Alternatively, it has been proposed that providing listeners with other-than-their-own HRTFs should affect their localization performance regardless of the saliency of spectral cues (Wenzel et al., 1993; Møller et al., 1996; Middlebrooks, 1999b). Four studies compared the localization performance obtained using the individual's own HRTFs (normal cues) to the performance obtained using non-individual HRTFs (modified cues) in the same listeners. The two studies involving listeners with previous experience in localization tests reported a difference in performance between HRTFs (Møller et al., 1996; Middlebrooks, 1999b). Conversely, the two studies involving naïve listeners reported no difference (Bronkhorst, 1995; Begault et al., 2001). The latter negative findings may have been due to the involvement of naïve listeners, who usually have more variable performance—perhaps due to differences in the speed of procedural learning (e.g., handling of the response device, Djelani et al., 2000; Majdak et al., 2010). There were multiple other methodological differences between the four studies^1^. Reports of a lack of difference in performance could also result from insufficiently large “inter-spectral distance” (ISD) between individual and non-individual HRTFs (as defined by Middlebrooks, 1999a). On the other hand, the reports of large differences might be explained merely by the fact that the listeners did not learn to use the cues provided by the non-individual HRTFs. Perceptual learning produces a recalibration of the audio-spatial map (Hofman et al., 1998; Carlile and Blackman, 2013). By simulating complete recalibration, Majdak et al. (2014) showed that using non-individual HRTFs should have a moderate impact on sound localization performance. However, they found that non-acoustical factors (attention, perceptual abilities) would be highly relevant for predicting sound localization performance.

Non-acoustical factors, such as perceptual processes, have been proposed to explain the large individual differences reported in studies about discrimination between front and rear sources (Wightman and Kistler, 1999) and about sound localization in noise (Andéol et al., 2011, 2013). The perceptual processes involved in the analysis of spectral cues (Drennan and Watson, 2001; Sabin et al., 2012) and sound localization accuracy with individual HRTFs (Majdak et al., 2010) were both found to improve with training in the auditory task. In the latter study, acoustical cues were kept constant but sensory (visual) feedback was provided during training. The resulting improvement in localization performance was assumed to reflect perceptual learning. However, increased exposure to the experimental environment (e.g., apparatus) and/or procedural learning (i.e., learning of the task contingencies) could have also contributed to the observed improvement.

In the present study, we assessed the contributions of acoustical and perceptual factors to sound localization abilities with virtual sources under experimental conditions that were chosen specifically to address the confounds present in previous studies—i.e., factors that could interfere with, or mask, the actual contribution of the factor investigated. Twenty naïve listeners were given procedural training prior to sound localization tests in “classical” conditions (anechoic environment, constant target/head distance, large range of azimuths and elevations). Acoustical and perceptual factors were separately manipulated, and the resulting effects on localization performance were assessed.

To investigate the role of acoustical cues, sound localization performance was measured with individual and non-individual HRTFs (normal and modified cues). We quantified the “spectral strength,” which is assumed to quantify the amount of spectral detail, of each HRTF (Andéol et al., 2013), and the ISD between individual and non-individual HRTFs. The following observations would be in favor of a substantial contribution of acoustical factors to sound localization abilities with virtual sources: a relationship between performance and spectral strength with individual HRTFs, a difference in performance between individual and non-individual HRTFs, and a relationship between this behavioral difference and the ISD between HRTFs.

The role of perceptual processes was investigated as follows. A subset of 15 listeners performed training to the sound localization task with individual HRTFs. Seven listeners received visual correct-answer feedback during training (test group) and eight received no feedback (control group). The amount of training-induced learning was assessed by comparing pre- and post-test performance. The persistence of learning was assessed by a follow-up post-test. In studies of perceptual training, it is often assumed that the training regimen elicits more efficient perceptual learning if correct-answer feedback is provided (Amitay et al., 2010), particularly for complex tasks (Garcia et al., 2013). For sound localization, it has even been suggested that no perceptual learning can occur if no feedback is provided (Recanzone et al., 1998; Irving and Moore, 2011). We therefore assumed that the training regimen in the present study elicited perceptual learning for the test group only. For this group, significant training-induced improvements in localization performance would indicate that perceptual learning occurred. The finding of a relationship between the amount of learning and the performance as measured prior to training for the test group would therefore reflect the contribution of a common—perceptual in this case—factor to the two behavioral metrics. Taken together, these results would indicate a large contribution of perceptual factors to sound localization abilities with virtual sources.

## Materials and methods

### Overview of the study

To test the hypotheses presented in the Introduction, two consecutive experiments were conducted. In the first experiment, the role of acoustical factors was assessed by comparing the localization performance obtained using individual HRTFs (normal acoustical cues) to that obtained using non-individual HRTFs (modified cues). The spectral strength of each HRTF, and the ISD between individual and non-individual HRTFs, were evaluated. Prior to the sound localization tests, each listener performed procedural training with visual targets to reduce the contribution of procedural factors to the results. The second experiment assessed the role of perceptual factors by comparing localization performance prior to and following a 5-day training regimen. A first group received visual feedback (test group) and a second group (control group) received no feedback. An improvement of performance for the first group would be in favor of a contribution of perceptual factors to sound localization abilities with virtual sources, because acoustical factors were constant during training. The control group allowed to assess the potential contribution of other factors (familiarization, procedural learning,…) to the observed training-induced improvements.

### Listeners

Twenty-five naïve listeners participated (11 females, mean age 27 ± 5 years; right-handed according to the Edinburgh Handedness Inventory, see Oldfield, 1971). All had normal hearing (thresholds of 15 dB HL or less at octave frequencies from 0.125 to 8 kHz) and normal otoscopy. None had history of auditory pathology. Written informed consent was obtained, in agreement with the guidelines of the Declaration of Helsinki and the Huriet law on biomedical research in humans. Listeners were paid 10 €/h for their participation. After completion of the study, the data from five listeners were excluded due to errors in the processing of their HRTFs (see below).

### Experimental apparatus

**Figure 1:**
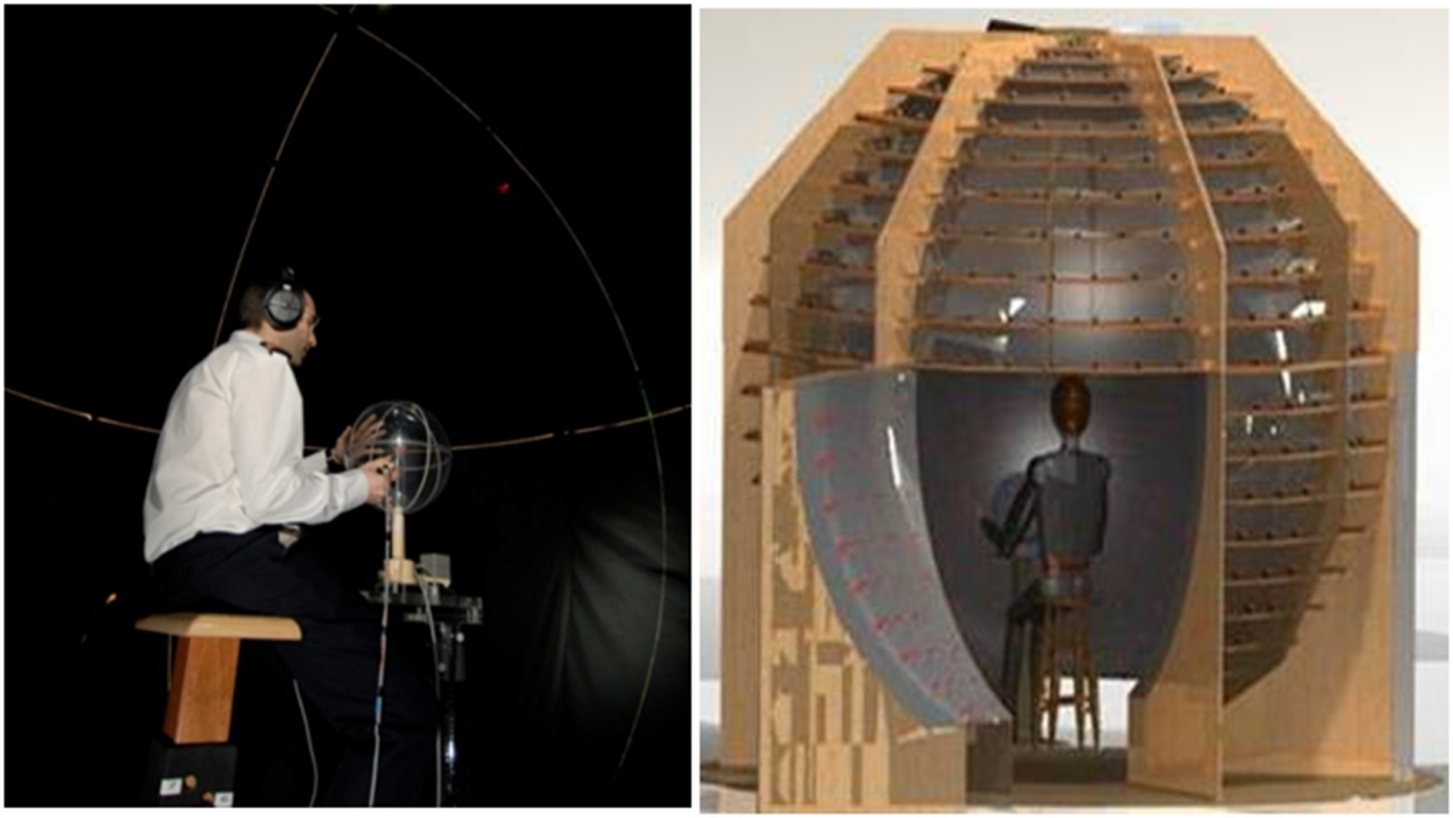
**Interior view (left) and exterior schematic view (right) of the experimental apparatus**.

The localization experiment was conducted inside a sphere, which was located in a 30-m^2^, light and sound-attenuating (<0.02 Lux and 35 dBA) room. The setup was a black sphere with a radius of 1.4 m that was truncated at its base (1.2 m below center, elevation = −60°). This sphere represented the perceptual space of the listener during testing (see Figure 1). Three lines of optical fibers were used to visually indicate the medial vertical, medial horizontal, and medial frontal planes on the interior surface of the sphere. A network of 619 optical fibers, each connected to one LED, was distributed on the sphere. The LEDs (color = red, size = 1° of visual angle, luminance = 10 cd/m^2^), when turned on, were used either as visual targets or as feedback signals.

The listener was seated on a stool that was adjusted so as to match the center of the listener's head with that of the sphere. During testing, the matching was verified using an electromagnetic sensor (Polhemus Fastrack) mounted on the headphones (Beyer DT990Pro). Listeners used a “God Eye Localization Pointing” system (GELP, Gilkey et al., 1995) to provide their localization responses. The GELP was composed of a plastic globe (radius = 15 cm) that represented a reduced version of the listener's perceptual space and a stylus. Listeners had to point the stylus on the globe so that the vector “center of the globe to stylus tip” had the same direction as the vector “center of the listener's head to perceived target direction on the sphere.” The position of the stylus tip was recorded using an electromagnetic sensor (Polhemus Fastrack), whose transmitter was mounted on the bar supporting the globe. To help the transfer of representation from perceptual to response spaces, the globe contained a figurine's head that represented the listener's head at the center of the sphere, and white circles that represented the three main planes (medial horizontal, medial vertical, and medial frontal). The position of the LEDs relative to the listener's head varied in azimuth from 0 to 360° and in elevation from −60 to 90°. The angular separation between LEDs was 15 or 20°.

### Measurement and spectral characterization of HRTFs

One non-individual (Neumann KU-100 dummy head) and 25 individual (listeners) HRTFs were measured in a semi-anechoic room (Illsonic Sonex Audio) using the procedure described in Andéol et al. (2013). Directional transfer functions (DTFs) were then derived from each HRTF using the method proposed by Middlebrooks (1999a). DTFs only contain the directional components of the HRTF, and are independent of the characteristics of the microphone or of its positioning into the ear canal. To compute DTFs, each HRTF has to be divided by the square root of the weighted sum of squared HRTFs that have been measured for each sound source direction. The weights are adjusted to take into account the non-uniform distribution of sound directions. The spectral strength, which corresponds to the ISD between a flat spectrum and the magnitude spectrum of the DTF, was computed for each HRTF using the procedure described in Andéol et al. (2013). The ISD between individual and non-individual HRTFs was quantified as the difference in DTF.

As a result of an error in DTFs computation (i.e., use of the HRTF measured for the 90° elevation instead of the weighted sum of squared HRTFs), which was detected after collection of the behavioral data, five listeners were excluded from the study. They had ISDs between correctly and incorrectly assessed DTFs greater than the smallest ISD between individual and non-individual HRTFs in the 25-listener cohort (9.5 dB^2^). ISDs between correct and incorrect DTFs ranged from 1.1 to 6.6 dB^2^ across the remaining 20 listeners (see Table 1). These values are below the ISDs between individual and non-individual HRTFs (range = 9.5 to 17.2 dB^2^). However, to verify that the error in DTFs was unlikely to affect the behavioral results reported below, five of the 20 listeners performed an additional localization test with individual HRTFs, using their correct and incorrect DTFs. The results showed little or no effect of the difference in DTF (see Appendix). We therefore refer below to “individual HRTFs” in spite of the small error in DTF presentation.

**Table 1:** **Individual value of the ISD between correct and incorrect DTFs (in dB^2^)**.

**Listener**	**ISD (dB^2^)**
L8	3.6
L9	4.4
L11	1.6
L12	1.4
L13	1.8
L14	2.5
L15	3.5
L17	3.9
L18	3.3
L21	2.2
L22	6.6
L23	1.3
L24	2.2
L26	4.9
L27	1.2
L28	2.7
L30	3.8
L31	4.1
L33	1.1
L34	1.3

### Stimuli

Stimuli for sound localization tests were digitally generated at a 48.8-kHz sampling rate, 24-bit resolution using a real-time processor (RX6 Tucker-Davis Technologies), and were converted to the analog domain, routed to a headphone buffer (HB7 Tucker-Davis Technologies) and presented through headphones (Beyer DT990Pro). The stimulus was a 150-ms (including 10-ms on/off cosine-squared ramps) burst of pink noise that was filtered between 0.05 and 14 kHz using sixth-order and seventh-order Butterworth filters, respectively. The overall stimulus level was 60 dB SPL.

### Procedures

Listeners (*N* = 20 after removal of five listeners) performed procedural training with the GELP using visual targets (3 consecutive days) and then completed sound localization pre-tests with individual and non-individual HRTFs in counterbalanced order (2 days). A subset of 15 listeners then performed training to the sound localization task with individual HRTFs (5 days) followed by sound localization “immediate” post-tests with individual and non-individual HRTFs in fixed order (2 days). All except one trained listeners performed a “long-term” post-test with individual HRTFs (1 month after the immediate post-tests).

The directions of the visual or auditory targets were chosen as follows. For sound localization tests, virtual auditory targets were created by interpolating the directions used for the HRTF measurement. The target directions were determined using 119-point meshes mapped onto the surface of the perceptual space (shortened at −60° of elevation) using the Hypermesh (Altair, MI, USA) software. Three different meshes were used for the pre-test, immediate post-test, and long-term post-test. A 7° azimuth translation was applied so that the directions tested using individual HRTFs were different from those tested using non-individual HRTFs. For the procedural and auditory trainings, the target directions corresponded to the positions of the optical fibers on the surface of the sphere. The surface of the sphere was divided into eight areas defined by the intersection of the median horizontal, vertical and frontal planes. For a given session of procedural or auditory training, the target directions were randomly but equally chosen among the eight areas. The target directions varied between sessions. Thus, the sets of 119 (sound localization tests) or 120 (auditory training) target directions varied between training sessions, between pre- and post-tests, and between individual and non-individual HRTFs.

#### Procedural training

The setup and response device were the same as those used for auditory tests. The procedural training stage had two goals: (1) familiarize the listener with the experimental environment and (2) reduce experimental noise related to the use of the response device (i.e., pointing errors in the transfer of representation from egocentric perceptual space to allocentric response space). Visual targets were used to prevent auditory learning.

Once the listener was installed in the sphere, a visual cross was turned on to indicate the “straight ahead” direction (azimuth and elevation = 0°). The listener oriented to the straight ahead direction and pressed the stylus button. The cross was turned off and a red visual target was then presented on the sphere by turning on one LED. For trials with no feedback, listeners had to indicate the perceived direction of the visual target using the GELP, and to validate their response by pressing the stylus button. For trials with feedback, listeners pointed to the perceived direction without pressing the stylus button. If the spherical angular error between actual and pointed directions was below the “permissible” error (=8° for day 1; = error measured for the last no-feedback block of the preceding day—2° for days 2 and 3), a “hit” sound was emitted. Otherwise, the listener had to modify the pointed direction until they reached permissible error. The trial ended either by the emission of the hit sound or after 30 s. The position of the target changed from trial to trial. The listeners performed three training sessions (duration = 1 h 30 each). For each session, two blocks of 40 trials with correct-answer feedback (15–20 min) alternated with three blocks of 32 trials with no feedback (12–15 min) in fixed order (no/with/no/with/no feedback).

The spherical angular error averaged across the 20 listeners decreased from 9.2° (±1.6) for the first to 6.6° (±1.3) for the last no-feedback blocks. Individual errors were stable across, at least, the last three no-feedback blocks (repeated measure ANOVA, error at no-feedback blocks as the within-listener factor, post-hoc Tukey-HSD: *p* > 0.50).

#### Sound localization tests

Before each presentation of the auditory target, the listener's position relative to the straight ahead direction was verified using the electromagnetic sensor. In case of a deviation above 5°, a message required the listener to rectify their position. Once the listener was correctly positioned, the auditory target was presented over headphones at one of 119 possible virtual directions on the sphere. The listener was free to move after the offset of the auditory target. The listener had to indicate the perceived direction using the GELP. There was no time restriction but listeners were encouraged to respond quickly. No correct-answer feedback was provided. The set of 119 directions was repeated six times (total number of trials = 714). The responses collected at the first repetition were excluded from the analyses. Each pre- and post-test had an overall duration of 1.5–2 h, and was divided into three series of four 60-trial blocks (54 for the last one). Listeners had to stay inside the sphere during between-block breaks (1.5 min) but were allowed to leave the setup during between-series breaks (10 min).

#### Auditory training

The auditory stimuli used during training had the same characteristics as those used in the sound localization pre- and post-tests except that only individual HRTFs were used. Each of the five training sessions included three 20-min blocks of 40 trials, with 8-min breaks between blocks. For the test group (*N* = 7), training consisted in providing the listener with trial-by-trial visual feedback (red LED turned on during 250 ms after the listener's response) as to the correct auditory target direction. Listeners were instructed to search for the red light, face it, and come back to the straight-ahead position. The auditory target + visual feedback sequence was replayed at least once. Listeners were then allowed to replay the sequence as many times as they wished. Training for the test group was similar to that used in the study by Majdak et al. (2010), except that their listeners were allowed only one sequence replay. For the control group (*N* = 8), training sessions were identical to pre- and post-tests sessions, except for the number of trials (660 trials instead of 714) that allowed the training duration to be similar for the two groups. The events and listener's actions during testing are listed in Table 2.

**Table 2:** **Order of events and listener's actions during auditory training**.

**Events**	**Listener's actions**
Straight ahead indicator turned on	Face the straight ahead indicator
Auditory target presentation	Indicate the target direction using GELP
Visual feedback (red light) turned on	Face the red light and come back
Straight ahead indictor turned on	Face the straight ahead indicator
Visual feedback turned off	
Auditory target re-presentation	
Visual feedback turned on	Choose to replay the auditory target + visual feedback sequence or to move to the next trial

### Data analysis

Localization responses were computed using a three-pole coordinate system (Kistler and Wightman, 1992). In this system, the position of a point is coded by the three following angles: the left/right angle in the medial vertical plane (direction in the left/right dimension), the front/back angle in the medial frontal plane (direction in the front/back dimension), and the up/down angle in the medial horizontal plane (direction in the up/down dimension). This coordinate system has the advantage that a given angular distance corresponds to a constant distance on the sphere for all spatial regions. Conversely, in two-pole—lateral/polar (Middlebrooks, 1999b) and azimuth/elevation (Oldfield and Parker, 1984)—coordinate systems, a compression of space occurs when points are close to the poles. Another advantage of the three-pole system is the distinction between spatial dimensions that depend on different localization cues or processes: binaural cues for localization in the left/right dimension (Strutt, 1907), spectral-shape analysis (Wightman and Kistler, 1993) or determination of the main spectral-notch position (Butler and Belendiuk, 1977) for localization in the up/down dimension, and comparison of the levels of different bandwidths (Wightman and Kistler, 1997) or more complex cues (Bronkhorst, 1995; Zhang and Hartmann, 2010) for localization in the front/back dimension.

**Figure 2:**
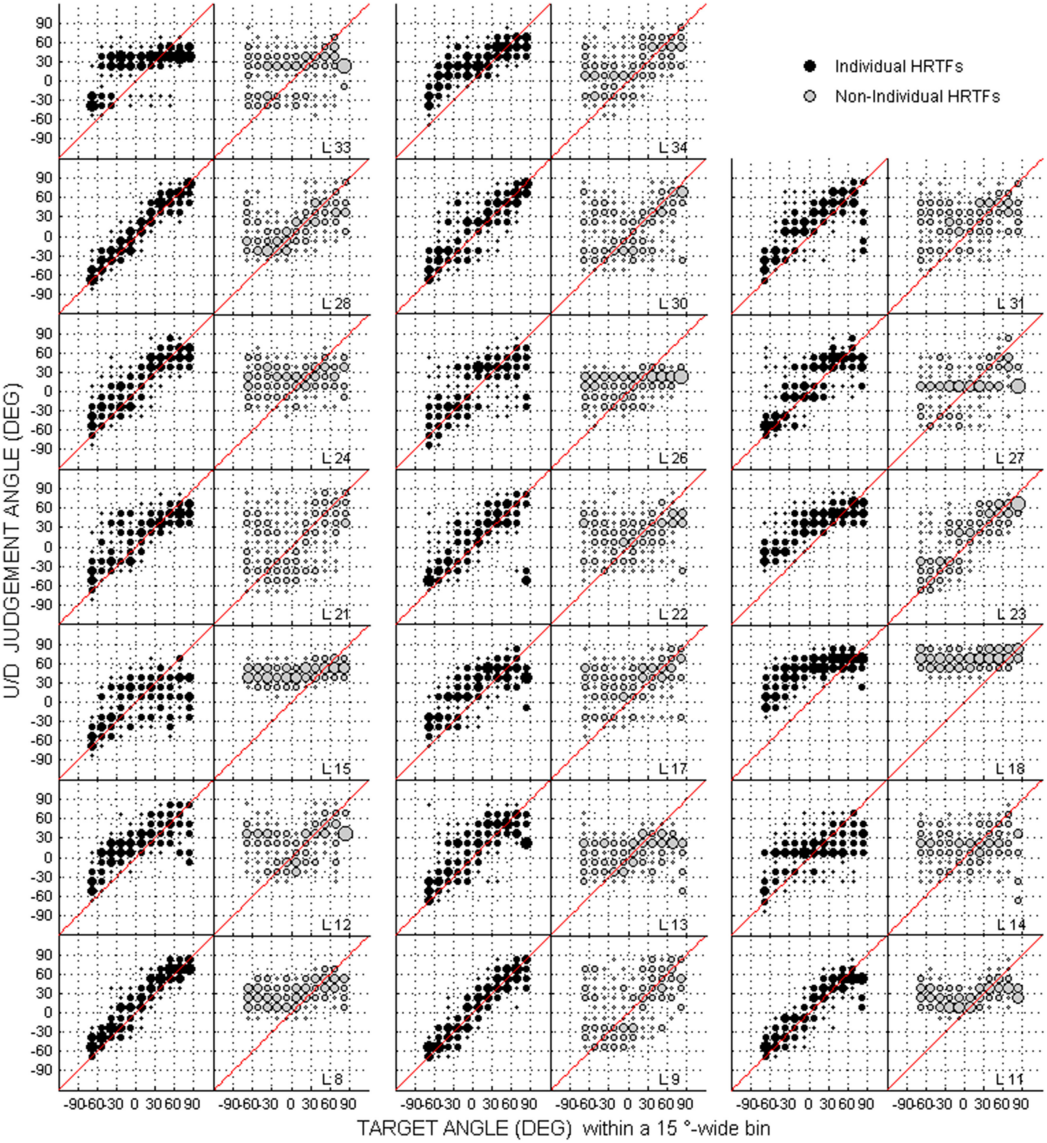
**Individual judgment position against target position with individual and non-individual HRTFs (black and gray dots, respectively) at the pre-test in the up/down dimension**. Each panel couple is for a different listener (*N* = 20).

**Figure 3:**
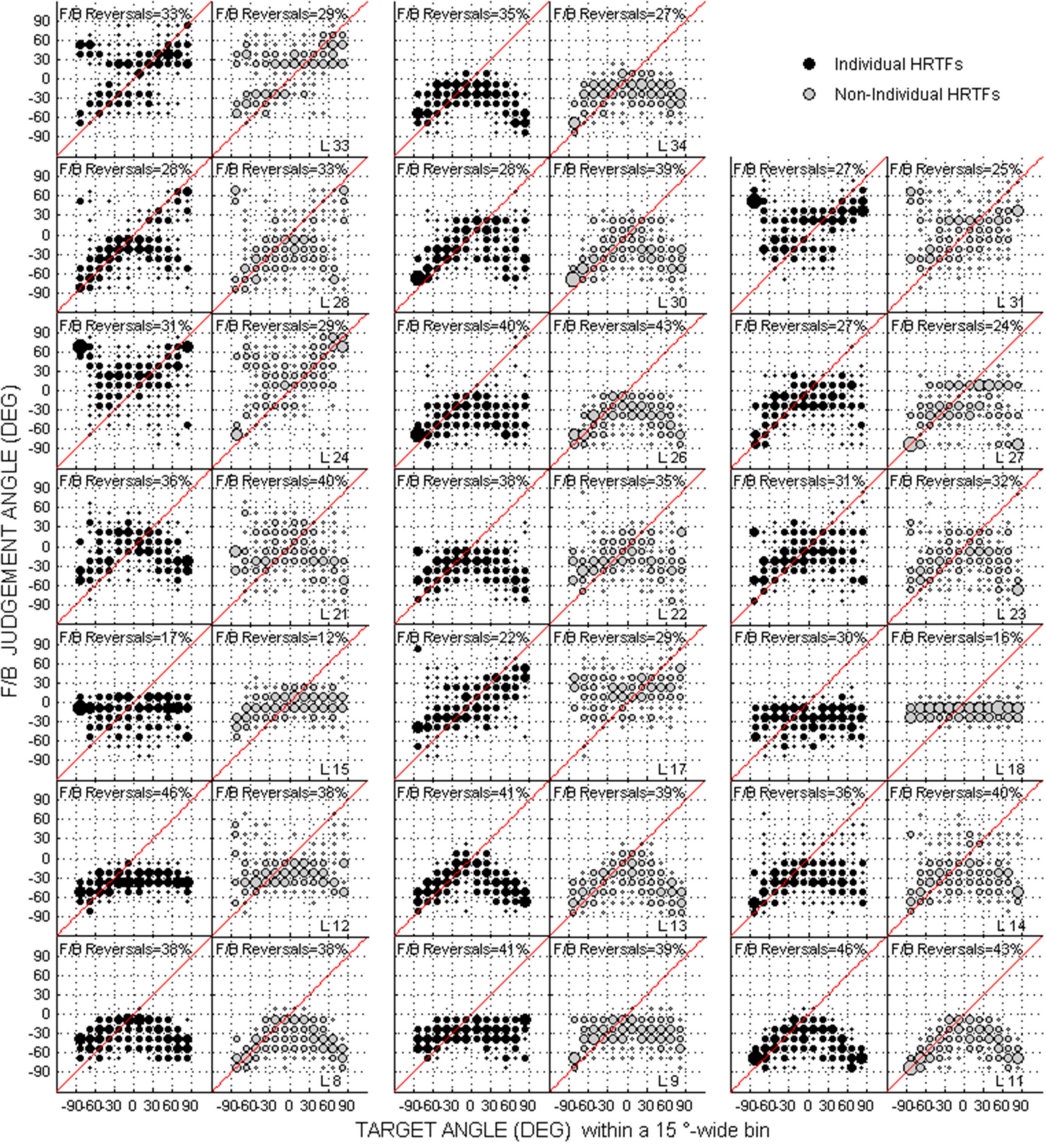
**Same as Figure 2 but for the front/back dimension**. The front/back reversal rate for individual and non-individual HRTFs are indicated in each panel couple.

**Figure 4:**
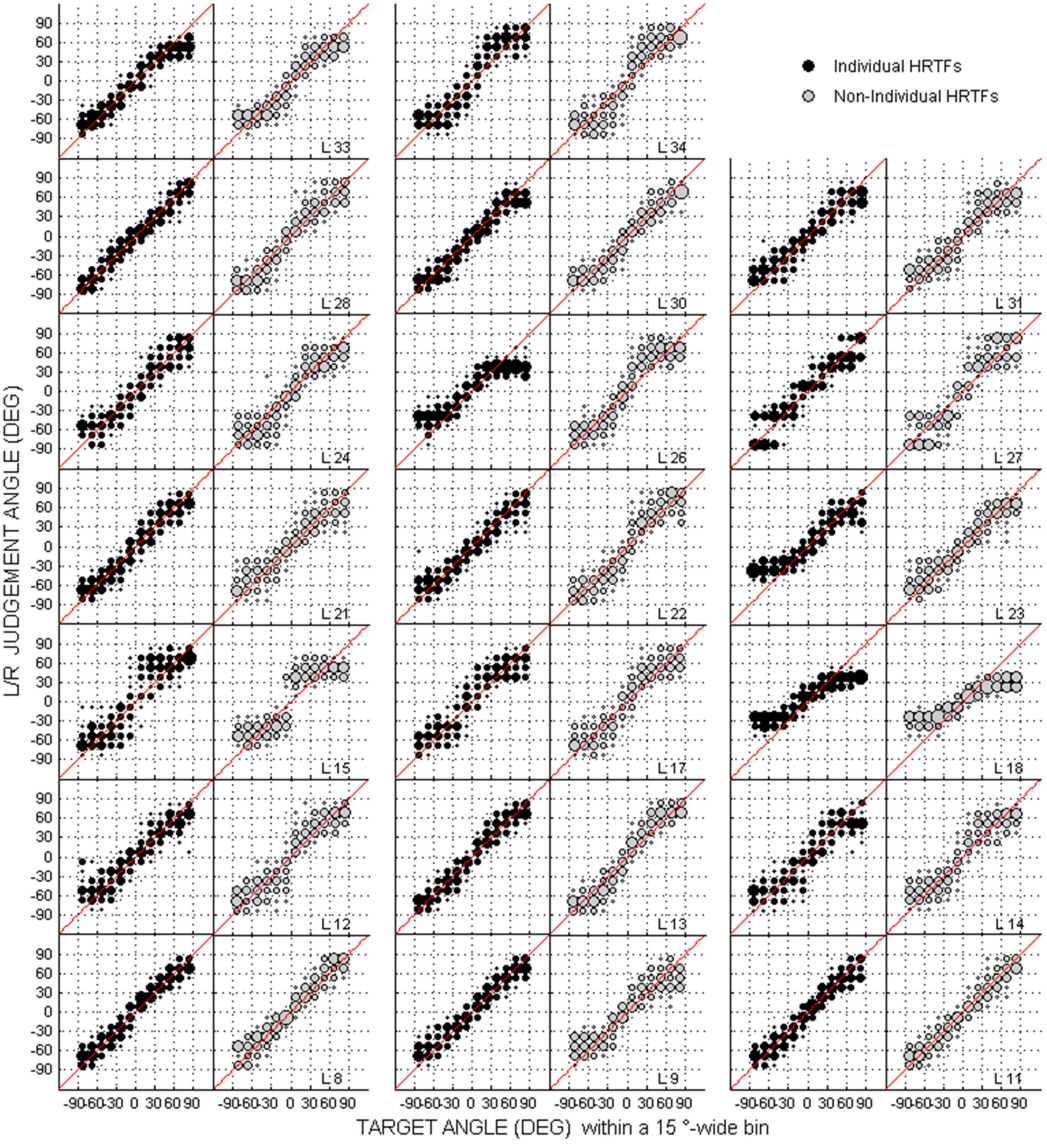
**Same as Figure 2 but for the left/right dimension**.

Scatterplots of raw data (i.e., target against response directions) are provided in Figures 2–4 for the up/down, front/back, and left/right dimensions, respectively. Because left/right judgments remain generally accurate with non-individual HRTFs (Wightman and Kistler, 1997), and individual differences in localization abilities were mainly observed for up/down and front/back dimensions, statistical analyzes were performed for the latter two dimensions only.

Numerous studies have reported frequent front/back (response pointing to the frontal hemifield for a target presented in the rear or vice versa) and up/down reversals (response pointing to above 0° elevation for a target presented at below 0° elevation or vice versa) in localization responses. Such reversals drastically increase angular errors, unless they are excluded or corrected (e.g., a response at −50° elevation is transformed into 50°). We therefore assessed the following localization scores: up/down angular error after correction of up/down reversals (in °), and down → up, up → down, and front/back reversal rates (in %). Up/down errors were separately assessed for “high,” “middle,” and “low” target elevations (elevation = 25 to 75°, −15 to 15°, −60 to −25°, respectively). Responses at ±15° front/back angles and those at ±20° up/down angles were not considered as front/back and up/down reversals, respectively.

The within- and across-listener paired comparisons listed below were statistically assessed using Wilcoxon tests. Relationships between two metrics were assessed using Spearman correlation coefficients. Two-tailed *p*-values are reported below.

To examine the role of acoustical factors, we assessed:

1. The relationship between spectral strength and pre-test performance with individual HRTFs for the 20-listener cohort.
2. The individual and cohort differences between individual and non-individual HRTFs in pre-test performance.
3. The relationship between this behavioral difference and the ISD between individual and non-individual HRTFs for the cohort.

To examine the role of perceptual factors, we first computed individual amounts of training-induced improvement (i.e., pre-test – post-test difference in score, referred to below as “learning amount”) with individual HRTFs. Then, we determined for each listener whether learning was significant using a Wilcoxon test (pre-test against post-test scores). Finally, we assessed within each trained group:

1. The relationship between learning amount at the immediate post-test and pre-test score.
2. Whether the listeners with significant learning at the immediate post-test had similar immediate and long-term post-test scores.

## Results

### Relationship between spectral strength and pre-test performance with individual HRTFs

**Figure 5:**
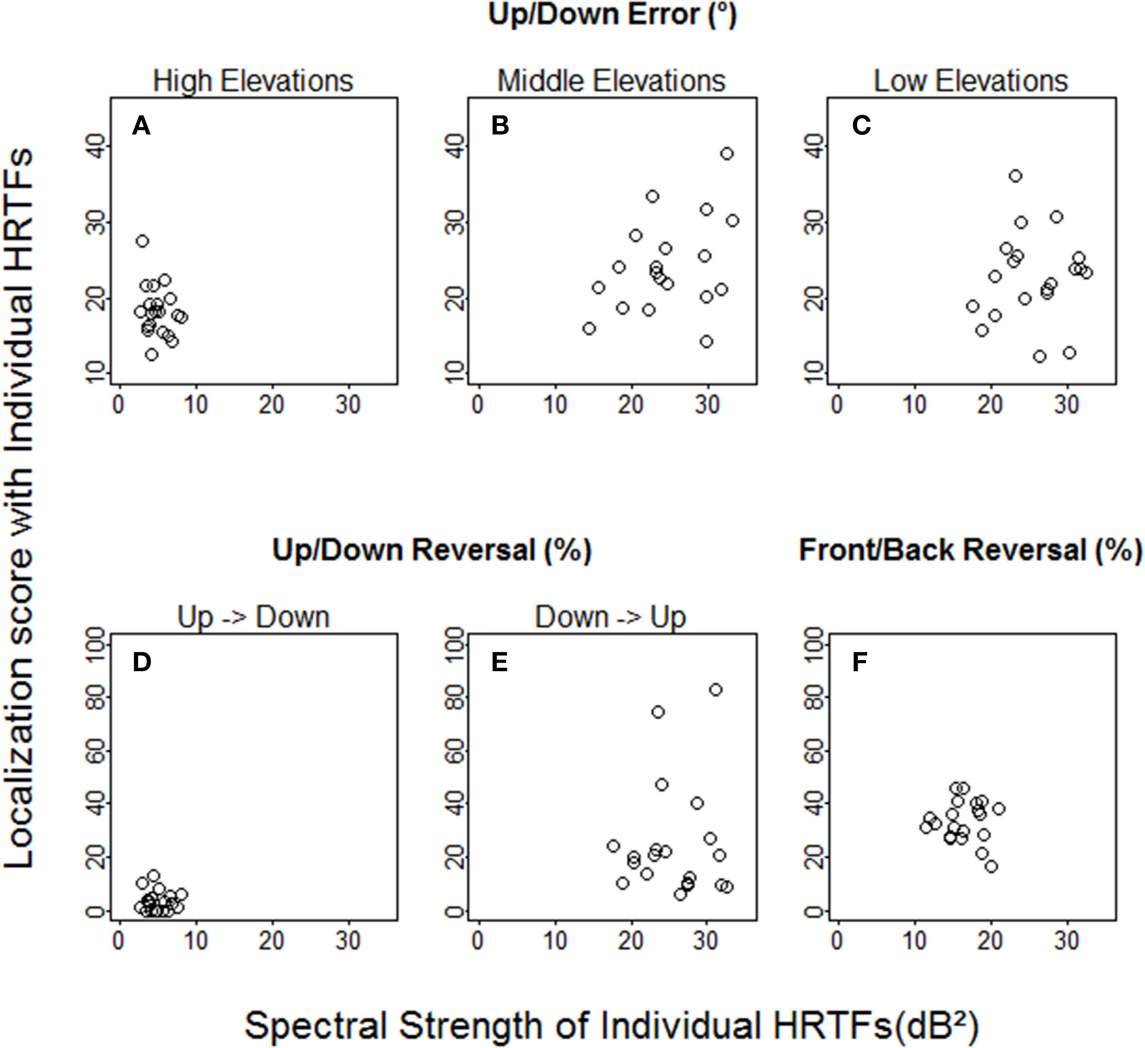
**Individual localization scores at the pre-test against spectral strength with individual HRTFs**. **(A–C)** Up/down errors (in °) for high, middle, and low target elevations. **(D–F)** Up → down, down → up, and front/back reversal rates (in %).

With individual HRTFs, no relationship was found between spectral strength and performance at the pre-test (see Figure 5), regardless of whether performance was expressed in terms of up/down angular errors (high elevations: *R* = −0.21, *p* = 0.37; middle elevations: *R* = 0.32, *p* = 0.16; low elevations: *R* = 0.14, *p* = 0.56), up/down reversals (up → down: *R* = −0.11, *p* = 0.64; down → up: *R* = −0.01, *p* = 0.95), or front/back reversals (*R* = −0.01, *p* = 0.99). However, the spectral strength of the non-individual HRTFs was weaker than that of all individual HRTFs (12.8 dB^2^ vs. 17.6 to 45.0 dB^2^) for the low elevation region, where (down → up) reversals were significantly more frequent with non-individual than with individual HRTFs.

### Difference between individual and non-individual HRTFs at the pre-test

**Figure 6:**
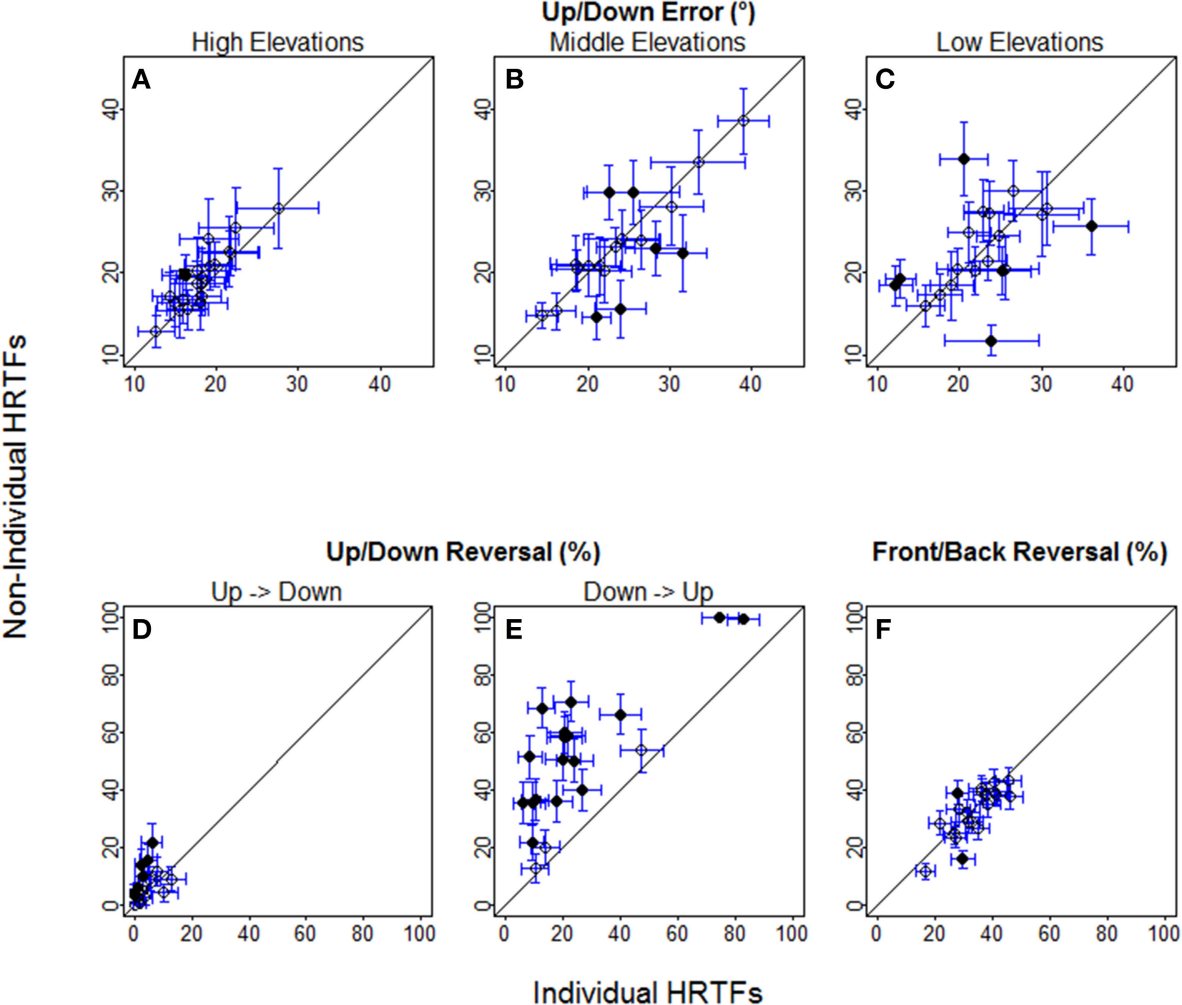
**Individual localization scores with non-individual against with individual HRTFs at the pre-test. (A–C)** Up/down errors (in °) for high, middle, and low target elevations. **(D–F)** Up → down, down → up, and front/back reversal rates (in %). Each symbol is for a different listener. Circles and bars represent the means and 95% confidence intervals averaged across about 30 (up/down error) to 96 (front/back reversals) target positions. Filled circles indicate the listeners with significant difference between individual and non-individual HRTFs according to Wilcoxon tests.

For up/down errors (see Figures 2, 6A–C), only a few listeners (1, 6, and 6 for high, middle, and low target elevations, respectively) individually showed significant differences between HRTFs. The lack of difference was observed regardless of whether listeners had large or small errors, and is therefore unlikely to have been due to a floor effect. The difference between HRTFs as assessed for the cohort was significant for high target elevations (median up/down error ± 1 inter-quartile range = 18 ± 3° with individual HRTFs < 19 ± 5° with non-individual HRTFs, *p* = 0.004) but was not significant for middle (24 ± 8° vs. 23 ± 8°, *p* = 0.52) and low target elevations (23 ± 6° vs. 21 ± 8°, *p* = 0.99). Up → down reversals were infrequent with individual HRTFs (see Figure 6D). The difference between HRTFs was small but significant for six listeners and for the cohort (median = 3 ± 5% with individual HRTFs vs. 5 ± 7% with non-individual HRTFs, *p* = 0.03). Down → up reversals were more frequent than up → down reversals, and increased with non-individual HRTFs (see Figure 6E). The difference between HRTFs was significant for 17 listeners and for the cohort (median = 20 ± 14% < 51 ± 26%, *p* < 0.001). For front/back reversals (see Figures 3, 6F), only two listeners individually showed significant difference between HRTFs. The difference for the cohort was not significant (median = 35 ± 10% ≈ 35 ± 11%, *p* = 0.37). Visual inspection of raw data in the left/right dimension indicates no difference between HRTFs (see Figure 4).

### Relationship between behavioral difference and ISD between individual and non-individual HRTFs

**Figure 7:**
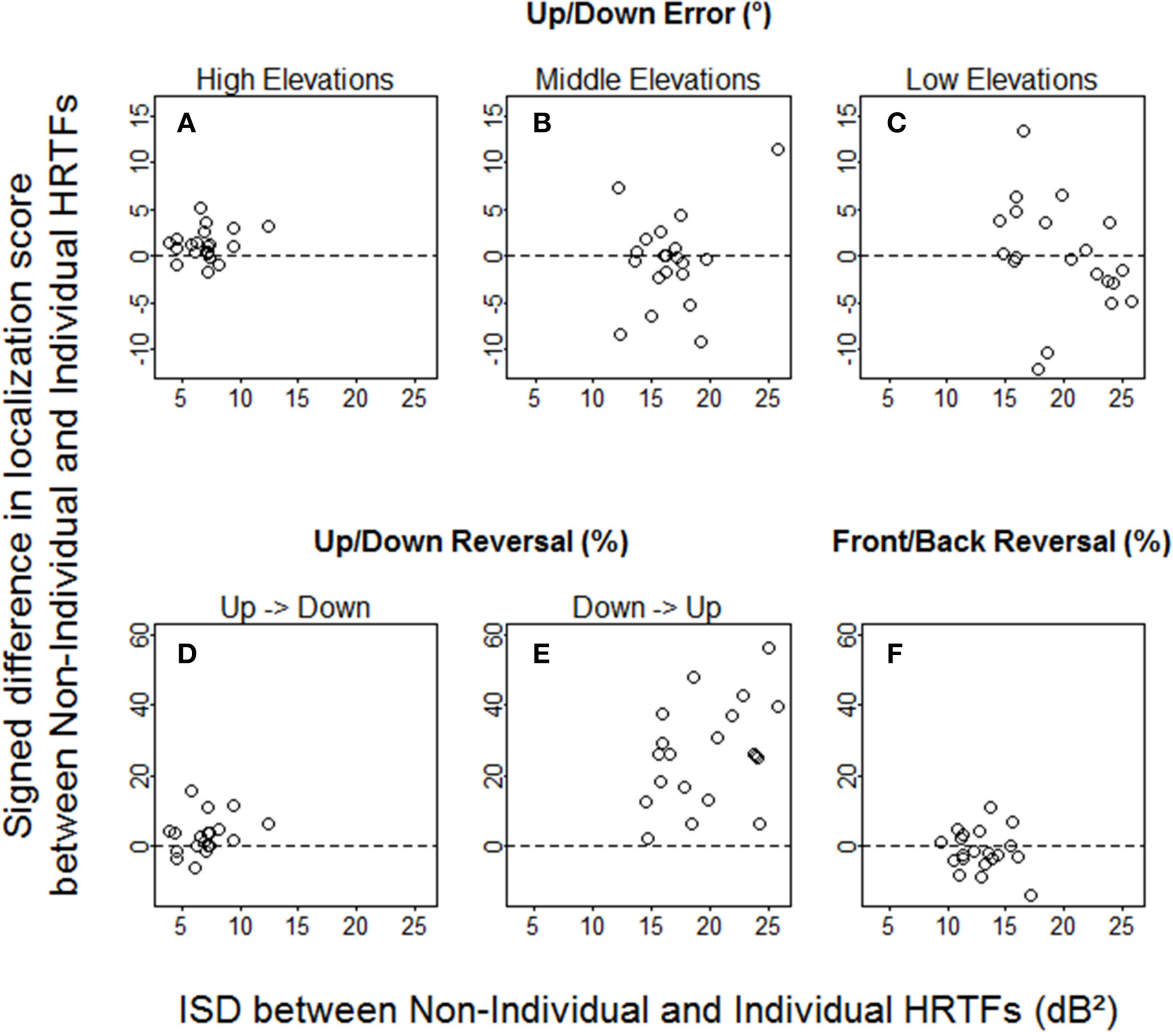
**Individual signed differences in localization score against ISD between non-individual and individual HRTFs**. **(A–C)** Up/down errors (in °) for high, middle, and low target elevations. **(D–F)** Up → down, down → up, and front/back reversal rates (in %).

The ISD values varied across target regions and listeners (Figure 7), but were essentially—except for high elevations—well-above 10 dB^2^, which should be large enough to produce behavioral effects according to the results from a past study (Middlebrooks, 1999b). However, we found no *positive* correlation between the signed difference in localization score and the ISD between non-individual and individual HRTFs (up/down errors: *R* = −0.03, *p* = 0.90 for high elevations, *R* = −0.07, *p* = 0.77 for middle elevations, *R* = −0.42, *p* = 0.037 for low elevations; up → down reversals: *R* = 0.32, *p* = 0.16; down → up reversals: *R* = 0.37, *p* = 0.11; front/back reversals: *R* = −0.02, *p* = 0.93). Note that if the listeners who had *lower* scores with non-individual HRTFs than with individual HRTFs were excluded from analyses, no correlation was significant.

### Significance of learning with individual HRTFs

**Figure 8:**
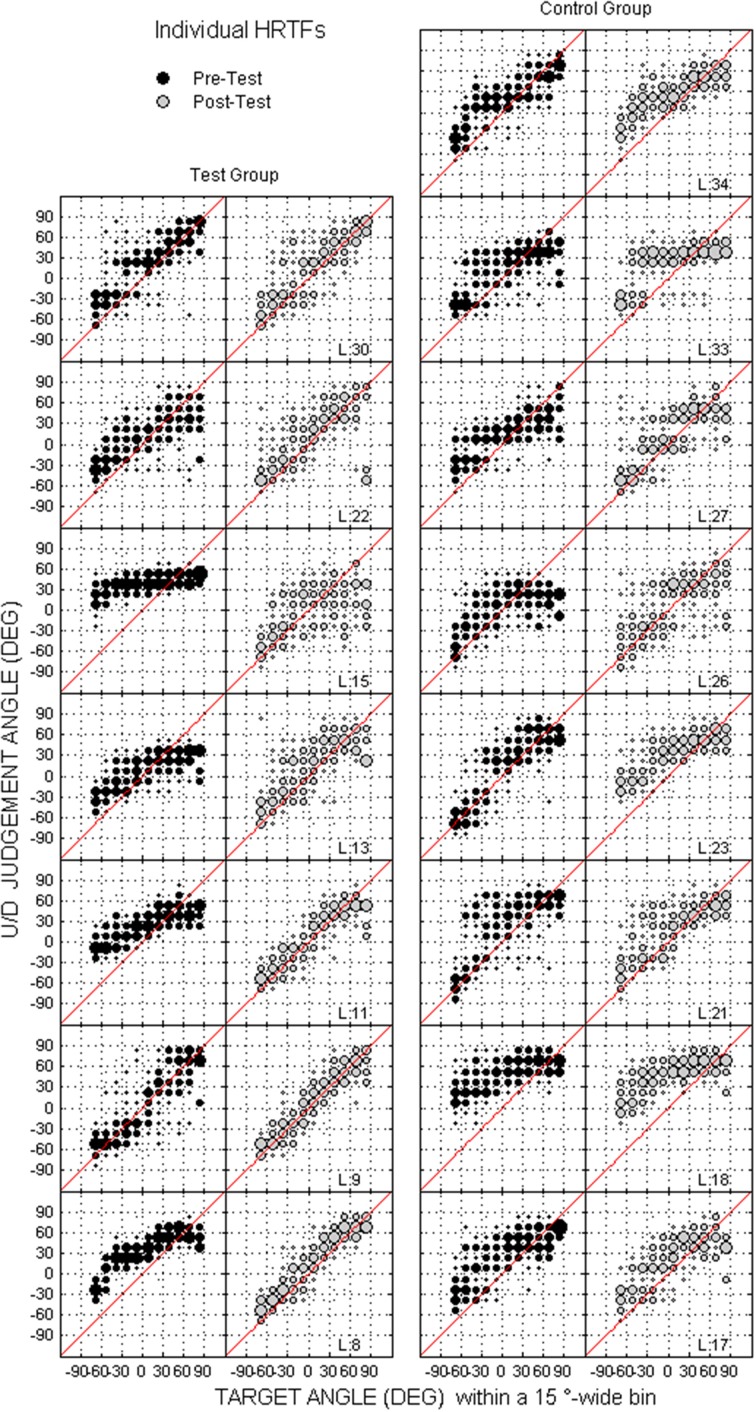
**Individual judgment position against target position with individual HRTFs at the pre- and post-tests (black and gray dots, respectively) for the test and control listeners (left and right columns, respectively) in the up/down dimension**. Each panel couple is for a different listener.

**Figure 9:**
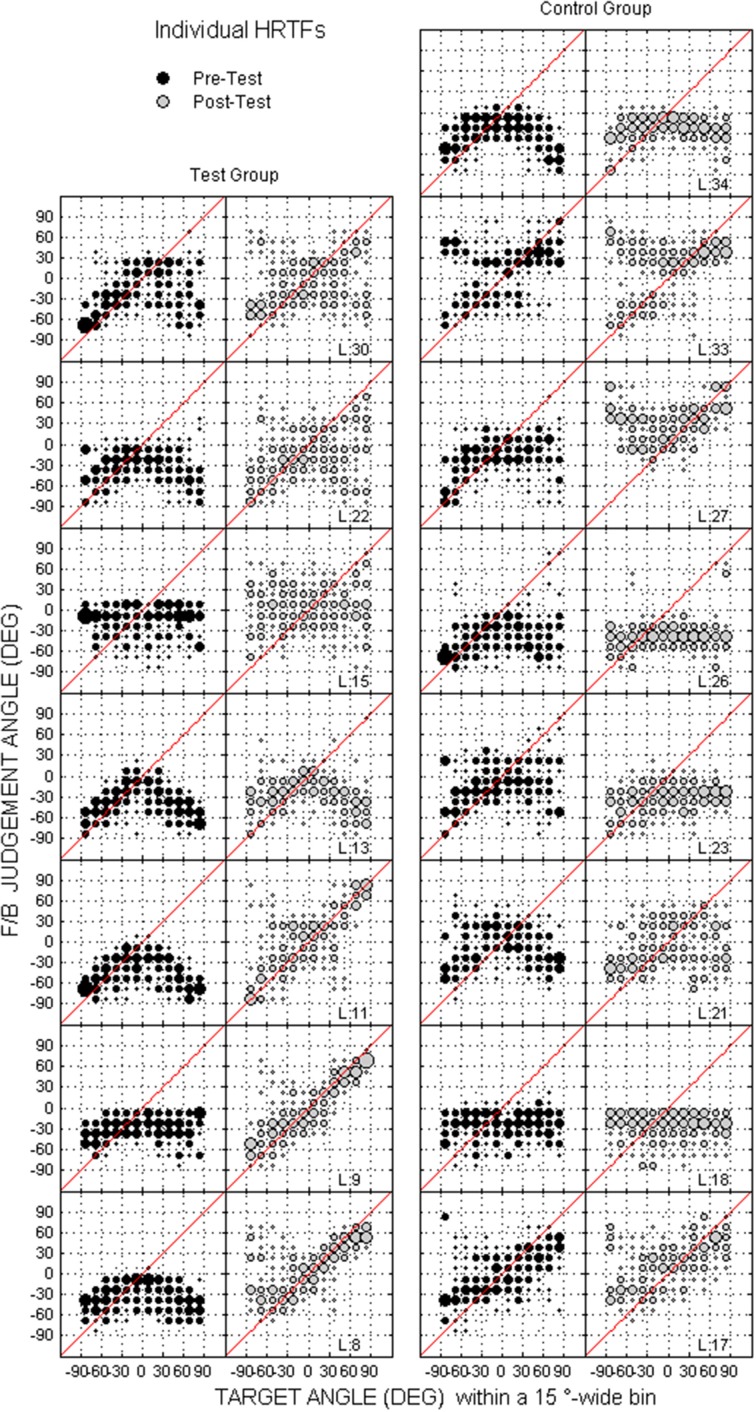
**Same as in Figure 8 but for front/back dimension**.

**Figure 10:**
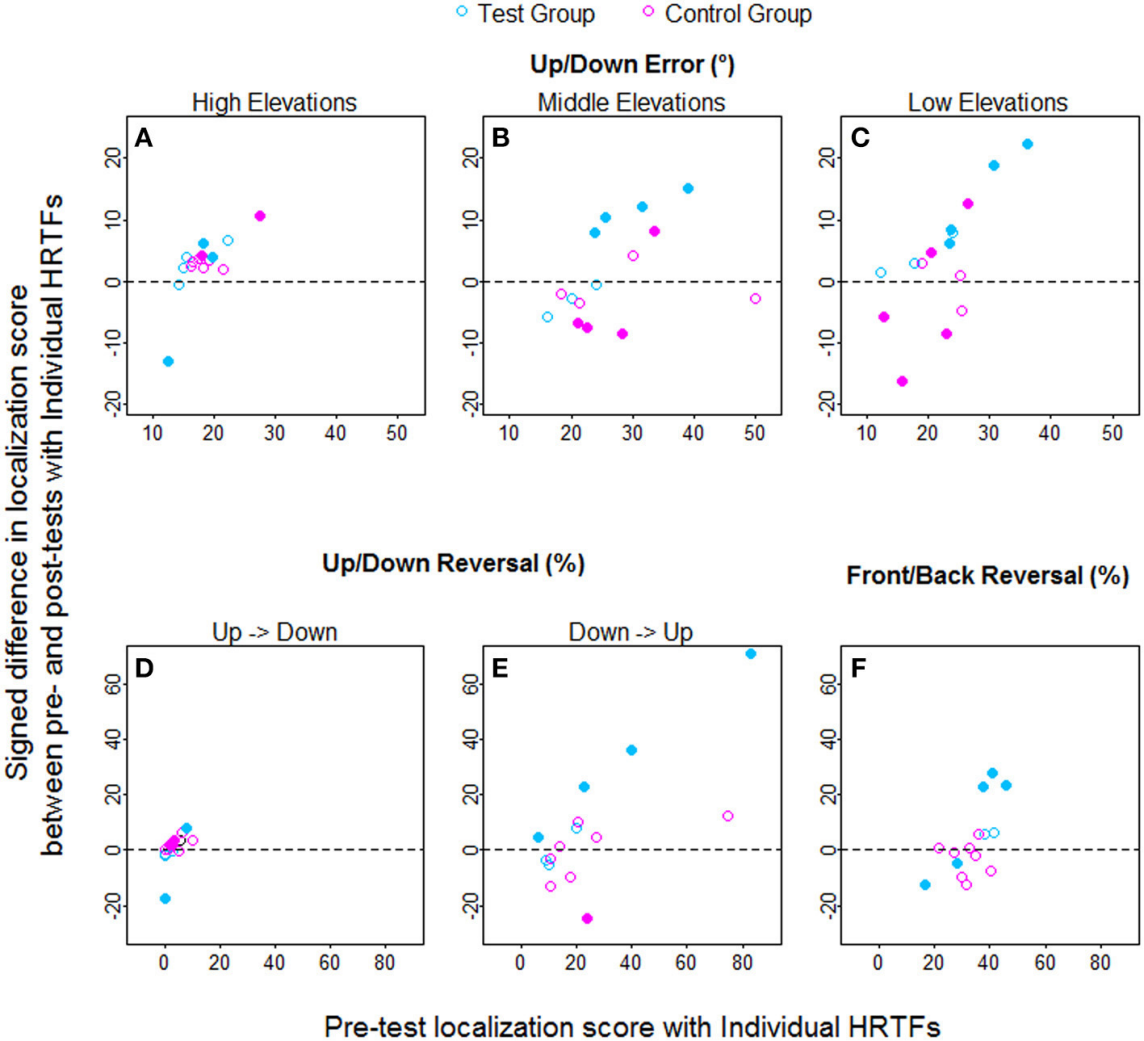
**Individual learning amounts (pre-test minus post-test localization score) against pre-test scores for the test and control listeners (blue and pink symbols, respectively) with individual HRTFs**. **(A–C)** Up/down errors (in °) for high, middle, and low target elevations. **(D–F)** Up → down, down → up, and front/back reversal rates (in %). Filled symbols indicate the listeners with significant difference between pre- and post-tests according to Wilcoxon tests.

Individual raw data collected at the pre-test and the post-test for the two groups are provided for the up/down and front/back dimensions in Figures 8, 9, respectively. In the up/down dimension, the listeners from the test group mostly showed substantial training-induced improvement in performance (i.e., post-test responses closer to perfect performance than pre-test responses, see left panels in Figure 8), but those from the control group showed little or no improvement (see right panels in Figure 8). For up/down errors, many listeners from the test group (2, 4, and 4/7 for high, middle, and low target elevations, respectively) but only a few listeners from the control group (2, 1, and 2/8, respectively) showed significant learning (see filled symbols above the dashed lines in Figures 10A–C). Up → down reversals were infrequent prior to training but nonetheless significantly decreased with training for one listener from the test group and for two listeners from the control group (see Figure 10D). Down → up reversals were frequent prior to training and significantly decreased with training for four listeners from the test group but for no listener from the control group (see filled symbols above the dashed line in Figure 10E). In the front/back dimension, post-test responses were similar to pre-test responses for all except one listener (L27) from the control group (see right panels in Figure 9), but frequently came closer to perfect performance with training for the test group, particularly for targets presented in front (see left panels in Figure 9). Learning as assessed on front/back reversal rates was significant for three listeners from the test group but for no listener from the control group (see filled symbols above the dashed line in Figure 10F).

At the pre-test, no significant difference was observed between the test and control groups (up/down errors: 16 ± 4° vs. 18 ± 2°, *p* = 0.28 for high elevations, 24 ± 6° vs. 25 ± 10°, *p* = 0.87 for middle elevations, 24 ± 7° vs. 22 ± 7°, *p* = 0.61 for low elevations; up → down reversals: 2 ± 4% vs. 3 ± 3%, *p* = 0.44; down → up reversals: 20 ± 22% vs. 19 ± 12%, *p* = 0.69; front/back reversals: 38 ± 8% vs. 32 ± 6%, *p* = 0.19). At the post-test, the test group had significantly smaller up/down errors for middle and low target elevations, and smaller down → up reversal rates, than the control group (22 ± 6° vs. 27 ± 7°, *p* = 0.004, 15 ± 3° vs. 21 ± 15°, *p* = 0.02, and 12 ± 9% vs. 23 ± 20%, *p* = 0.01, respectively). However, no significant between-group difference was observed in up/down errors for high target elevations and in up → down reversals (15 ± 3° vs. 15 ± 2°, *p* = 0.54 and 2 ± 2% vs. 0.3 ± 2%, *p* = 0.17, respectively).

### Relationship between learning amount and pre-test results with individual HRTFs

The correlations between learning amount and pre-test score were assessed for each variable and group. For up/down errors, learning significantly increased with the pre-test score for the test group (*R* = 0.96, *p* = 0.003 for all target elevations), whereas no correlation was found for the control group (*R* = 0.14, *p* = 0.75; *R* = 0.31, *p* = 0.46; *R* = 0.50, *p* = 0.22 for high, middle, and low elevations, respectively). For up/down reversals, the correlations were significant for the test group (up → down: *R* = 0.93, *p* = 0.003; down → up: *R* = 0.98, *p* < 0.001) but were not for the control group (up → down: *R* = 0.55, *p* = 0.17; down → up: *R* = 0.49, *p* = 0.22). For front/back reversals, no correlation was significant (test group: *R* = 0.75, *p* = 0.07; control group: *R* = −0.02, *p* = 0.98).

Furthermore, to check whether the improvement in performance reflected or not an adaptation to errors in DTF computation (see Section Measurement and Spectral Characterization of HRTFs), the correlations between learning amount and ISD between correct and incorrect DTFs were assessed. No *positive* correlation was found for any variable and group (test group: *R* = 0.07, *p* = 0.91; *R* = −0.07, *p* = 0.91; *R* = −0.79, *p* = 0.048 for high, middle, and low elevations, respectively. *R* = 0.68, *p* = 0.11; *R* = −0.29, *p* = 0.56; *R* = −0.07, *p* = 0.91 for up → down, down → up, and front/back reversals, respectively. Control group: *R* = −0.16, *p* = 0.71; *R* = 0.30, *p* = 0.47; *R* = 0.01, *p* = 0.98 for high, middle, and low elevations, respectively. *R* = 0.20, *p* = 0.63; *R* = 0.61, *p* = 0.11; *R* = −0.08, *p* = 0.84 for up → down, down → up, and front/back reversals, respectively).

### Retention of learning with individual HRTFs

All listeners with significant learning at the immediate post-test showed no significant difference in score between immediate and long-term post-tests (3/3 in the test group for down → up reversals and 2/2 in the control group for up → down reversals; 1/1, 3/3, and 3/3 in the test group and 2/2, 1/1, and 2/2 in the control group for up/down angular errors for high, middle and low elevations, respectively; 2/2 in the test group for front/back reversals).

## Discussion

### Role of acoustical factors

To examine the contribution of acoustical factors to sound localization abilities with virtual sources, we assessed for 20 naïve listeners the relationship between the spectral strength and the localization performance with individual HRTFs, the difference in performance between individual and non-individual HRTFs (normal and modified cues), and its relationship with the ISD between HRTFs. Localization performance was measured in terms of up/down angular errors following correction of reversals for three target elevations (high, middle, low), up → down reversals, down → up reversals, and front/back reversals rates. We found no relationship between spectral strength and performance with individual HRTFs, nor between behavioral difference and ISD between HRTFs. The only sizeable difference in performance between HRTFs appeared in the low elevation region. In that region, where the acoustical differences between HRTFs (in terms of spectral strength and ISD) were the largest, we noted that the target was perceived in the lower (i.e., correct) hemisphere with individual HRTFs but in the upper (i.e., incorrect) hemisphere with non-individual HRTFs. Past studies involving trained listeners found sizeable differences in localization performance between individual and non-individual HRTFs in both front/back and up/down dimensions (Møller et al., 1996; Middlebrooks, 1999b). Those involving naïve listeners reported little or no difference in the front/back dimension (Bronkhorst, 1995; Begault et al., 2001), as for the present study, but they also reported no difference in the up/down dimension, contrary to the present study.

Concerning the front/back dimension, the present findings indicate that the lack of difference in past studies was unlikely due to a floor effect in the (poor) performance of listeners with no prior experience in the task (Bronkhorst, 1995), or to an insufficient ISD between individual and non-individual HRTFs (Middlebrooks, 1999b). First, our listeners performed procedural training prior to auditory tests, which prevented exposure to the experimental environment and response device from affecting the results. Second, the lack of behavioral difference between HRTFs in the auditory task was observed regardless of whether the listener had good or poor performance. Third, most values of ISD between individual and non-individual HRTFs were assumed to be sufficiently large to affect behavioral results according to the results from a past study (Middlebrooks, 1999b).

Front/back reversal rates were substantially higher in the present study using individual HRTFs than in free-field past studies (Wightman and Kistler, 1989; Carlile et al., 1997; Martin et al., 2001). Higher front/back reversal rates for virtual sources presented with individual cues than for real sources have previously been reported (Wightman and Kistler, 1989; Middlebrooks, 1999b). These difference could possibly result from headphone transfer function issues (Wightman and Kistler, 2005), degree of spatial resolution during the HRTF measurement, and/or errors in DTF computation (present study, see Section Measurement and Spectral Characterization of HRTFs). In the present study, the error in DTF computation was present in both individual and non-individual HRTFs, and could therefore have reduced the behavioral differences between HRTFs.

Concerning the up/down dimension, the discrepancy between the present study and Bronkhorst (1995) and Begault et al. (2001) studies could arise from methodological issues. Bronkhorst used other listeners' HRTFs as non-individual HRTFs. Given our observations, this has probably reduced the differences in spectral strength—and therefore the behavioral differences—between individual and non-individual HRTFs. In the Begault et al. (2001) study, the auditory target positions were limited to the horizontal plane, excluding the low elevation region where we observed the strongest difference between individual and non-individual HRTFs.

We also suggested that the discrepancy between the four past studies (Bronkhorst, 1995; Møller et al., 1996; Middlebrooks, 1999b; Begault et al., 2001) could arise from differences in experimental protocol (see Footnote 1). In the present study, we used a “classical” protocol, which resembles the protocol used in a past study that reported a difference between HRTFs (Middlebrooks, 1999b). Beyond differences in the listener's characteristics (naïve in the present study but trained in the past study), we explain the discrepancy between the present and Middlebrooks's studies in terms of data analysis. Middlebrooks assessed reversals without distinction between the up/down and front/back dimensions, and angular (polar) errors following correction of reversals using a more conservative criterion than ours.

To sum-up, the lack of correlation between spectral strength and performance with individual HRTFs showed that this acoustical factor is not a good predictor of performance. Another acoustical factor is the degree of matching between the listener's individual localization cues and those provided by the signal to localize. Our results suggest that large mismatch is needed to produce behavioral effects. However, the validity of this statement is limited by the remaining uncertainty in the quality of the HRTFs.

### Role of perceptual factors

To examine the contribution of perceptual factors to sound localization abilities with virtual sources, a subset of 15 listeners performed training to the sound localization task with fixed acoustical cues (individual HRTFs). The listeners were provided with either sensory (visual) or no correct-answer feedback. We expected the training regimen to elicit perceptual learning, that is, an improvement in the perceptual processes involved in the analysis of acoustical cues, for the “test” group who received feedback. Beyond the use of feedback, the perceptual and procedural contributions to training-induced improvements in performance are rarely separated (Robinson and Summerfield, 1996; Wright and Fitzgerald, 2001). In the present study, the improvement observed following auditory training was unlikely to be triggered by procedural learning for several reasons. First, the listeners performed procedural training with non-auditory stimuli over 3 days prior to sound localization tests, which resulted in optimal and steady ability to handle the response device. Second, further exposure to the procedural aspects of the task during auditory training resulted in significant improvements for only a few listeners from the control group. Third, individual differences in learning amount were larger in the present study (see Figure 10) than those reported for procedural learning in a past study (training to interaural time and level differences, Wright and Fitzgerald, 2001). In addition, we observed that the training-induced improvements were retained after 1 month. This suggests that the improvement was not due to modification of the listening strategy, or to a temporary increase in the listener's attentional resources (Goldstone, 1998).

It could seem counter-intuitive that an improvement in sound localization performance is still possible despite a lifetime of localization learning. However, training-induced improvements with normal cues and correct-answer feedback have been reported in previous studies, including for the “most robust” localization ability (i.e., localization of real sources in the left/right dimension, see Savel, 2009; Irving and Moore, 2011). Moreover, improvements in the front/back dimension could result from increased weighting of spectral cues but decreased weighting of dynamic cues—available in everyday life conditions but unavailable in the present experiment (Wightman and Kistler, 1999)—to front/back discrimination following training. Part of the training-induced improvement observed with individual HRTFs could result from exposure to abnormal cues (i.e., incorrect DTFs). In agreement, there are multiple reports of learning of—adaptation to—abnormal spectral cues with exposure (Hofman et al., 1998; Van Wanrooij and Van Opstal, 2005; Carlile and Blackman, 2013). However, the ISD between normal and abnormal spectral cues (i.e., between correct and incorrect DTFs, see Table 1 and Appendix) in the present study was probably too small to produce significant improvement (Van Wanrooij and Van Opstal, 2005). Moreover, no positive correlation was found between the amount of improvement and the ISD between correct and incorrect DTFs.

Our findings confirm the results of a previous study that reported substantial improvement in sound localization with individual HRTFs after a similar training protocol (Majdak et al., 2010). Our results indicate furthermore that this improvement might not be explained by procedural learning.

As perceptual learning is often stimulus-specific, findings of a generalization of learning to untrained stimuli or conditions are mostly believed to reflect task or procedural learning (Wright and Zhang, 2009). However, it has been suggested that generalization could also reflect perceptual learning (Ahissar, 2001). In this case, the learning involves—often high level—sensory processes that are not specific to the task. In the present study, we assessed whether the listeners from the test and control groups who showed significant learning following auditory training in the trained condition (individual HRTFs) also showed significant learning in an untrained condition (non-individual HRTFs). No learning generalization was observed for the localization responses in the front/back dimension, but most listeners from the test group showed generalization for up/down reversals and up/down errors. Because these listeners had received procedural training, we assume that the generalization was perceptual. The generalization observed could mean that the training improved sensory processes that are not specific to sound localization with individual HRTFs. One of these processes could be, for example, the analysis of the spectral shape of the stimulus (Andéol et al., 2013), a process that is involved regardless of the HRTFs set. Overall, the results indicate that training-induced modifications of perceptual processes had substantial effects on localization performance with virtual sources.

Moreover, we found that the training-induced learning amount was related to the pre-training performance (i.e., poorer initial performance led to larger learning amount), a result also observed in several previous studies (Wright and Fitzgerald, 2001; Amitay et al., 2005; Astle et al., 2013). This correlation is in favor of a contribution of common—here perceptual—factors to the two metrics. In other words, our results suggest that perceptual processes account for individual differences in sound localization abilities with virtual sources in naïve listeners.

Taken together, these results are consistent with a large contribution of perceptual processes to sound localization abilities with virtual sources. Majdak et al. (2014) recently reached a similar conclusion using a sound localization model. By modifying model parameters relative to acoustical or non-acoustical factors, they found that non-acoustical factors (such as for example perceptual abilities to process localization cues) were better predictors of performance than acoustical factors (quality of the directional cues in the HRTFs).

## Conclusion

The study assessed the contributions of acoustical and perceptual factors to the ability to localize virtual sound sources presented in quiet for naïve normal-hearing young adults. The spectral strength of the HRTFs did not seem to be a relevant acoustical factor to account for localization performance. Only large modifications of acoustical localization cues seemed to produce behavioral effects, although technical issues with the normalization of the HRTFs might have blurred part of the results. Auditory training with visual correct-answer feedback and constant acoustical cues substantially improved performance. These findings are consistent with a greater role of perceptual factors than of acoustical factors in sound localization abilities with virtual sources. Further research is needed to assess whether the present results generalize to the case of localization in free field.

### Conflict of interest statement

The authors declare that the research was conducted in the absence of any commercial or financial relationships that could be construed as a potential conflict of interest.

## Figures and Tables

**Figure A1:**
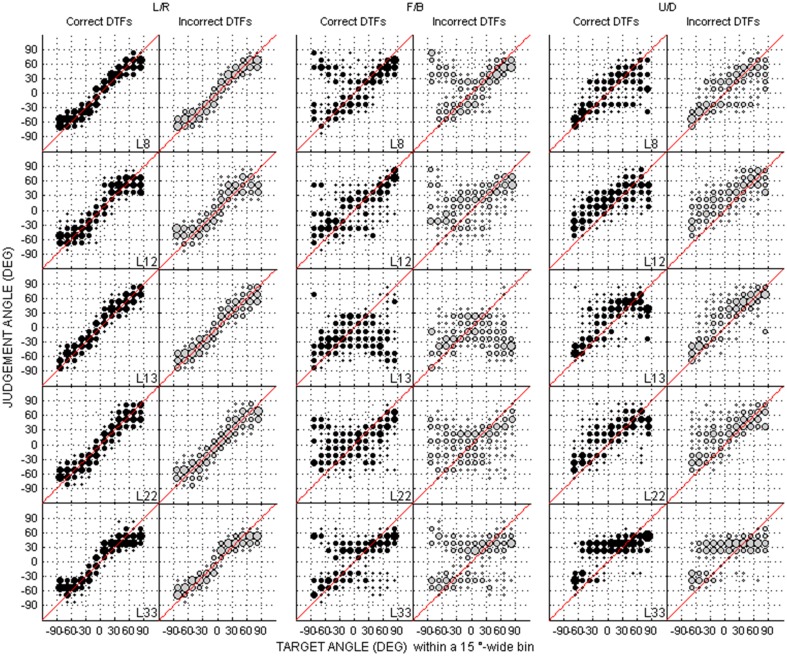
**Individual judgment position against target position using correct and incorrect DTFs (black and gray dots, respectively) with individual HRTFs in the left/right, up/down, and front/back dimensions**. Each panel couple is for a different listener (*N* = 5).

## Footnotes

^1^Middlebrooks (1999b) used a “classical” protocol with an absolute localization task, a virtual sound source simulated in an anechoic environment, a large range of source elevations and azimuths, and constant target/listener distance. Møller et al., (1996) used a non-anechoic environment and variable target distances. Bronkhorst (1995) used a forced-choice localization task. Begault et al. (2001) restrained the target positions to the horizontal plane.

## Appendix

An error in DTFs computation was detected following collection of behavioral data. To assess whether this error influenced behavioral results, we compared the performance with individual HRTFs obtained using correct DTFs to that obtained using incorrect DTFs in five listeners. The methods were similar to those used to compare individual and non-individual HRTFs (set of 119 target positions, six repetitions) except that the type of DTFs (correct or incorrect) randomly changed from trial to trial. Each listener performed 1428 trials over 2 days. The first 119 trials of each day, which contained approximately the same number of trials with correct and with incorrect DTFs, were excluded from the analyses. Visual inspection of the raw data in the left/right, front/back, and up/down dimensions showed similar results for correct and incorrect DTFs for each listener (Figure A1), including listener L22 who had the highest ISD between DTFs (6.6 dB^2^). Wilcoxon tests showed better performance with correct than with incorrect DTFs for only one of 30 comparisons (5 listeners × 6 variables, see Table A1): listener (L22) for up/down errors for high elevations (17° *vs*. 13°, *p* = 0.005). The differences between DTFs for the 5-listener group were not significant (up/down error: 17 ± 2° *vs*. 15 ± 3°, *p* = 0.06; 26 ± 7° *vs*. 26 ± 4°, *p* = 0.19; 19° ± 4 *vs*. 19 ± 6°, *p* = 0.63 for high, middle, and low target elevations, respectively; up → down reversals: 2 ± 01% *vs*. 2 ± 3%, *p* = 0.99; down → up reversals: 13 ± 9% *vs*. 19 ± 14%, *p* = 0.58; Front/back reversals: 38 ± 8% *vs*. 32 ± 6%, *p* = 0.19).

**Table A1:** **Comparison between correct and incorrect DTFs for each variable and each listener**.

			**L22**	**L8**	**L13**	**L12**	**L33**
Spectral strength of the individual HRTFs (dB^2^)		Incorrect DTFs	21.0	18.3	15.6	15.4	12.8
		Correct DTFs	15.6	15.2	13.3	14.0	11.9
Inter-DTF (Incorrect − Correct) ISD (dB^2^)			6.6	3.6	1.8	1.4	1.1
Up/down error (°)	High elevations	Incorrect DTFs	17	22	15	18	16
		Correct DTFs	13	19	14	18	15
		Difference	P = 0.005	*ns*	*ns*	*ns*	*ns*
	Middle elevations	Incorrect DTFs	26	22	29	21	29
		Correct DTFs	26	22	29	25	29
		Difference	*ns*	*ns*	*ns*	*ns*	*ns*
	Low elevations	Incorrect DTFs	20	16	15	29	19
		Correct DTFs	22	15	15	29	19
		Difference	*ns*	*ns*	*ns*	*ns*	*ns*
Up → down reversals (%)		Incorrect DTFs	3	9	2	1	2
		Correct DTFs	1	12	2	0	4
		Difference	*ns*	*ns*	*ns*	*ns*	*ns*
Down → up reversals (%)		Incorrect DTFs	19	3	13	11	35
		Correct DTFs	24	3	10	19	35
		Difference	*ns*	*ns*	*ns*	P = 0.030	*ns*
Front/back reversals (%)		Incorrect DTFs	26	24	30	19	32
		Correct DTFs	30	21	32	24	32
		Difference	*ns*	*ns*	*ns*	*ns*	*ns*
