# Corrigendum: Perceptual factors contribute more than acoustical factors to sound localization abilities with virtual sources

**DOI:** 10.3389/fnins.2016.00363

**Published:** 2016-08-08

**Authors:** Guillaume Andéol, Sophie Savel, Anne I. Guillaume

**Affiliations:** ^1^Département Action et Cognition en Situation Opérationnelle, Institut de Recherche Biomédicale des ArméesBrétigny sur Orge, France; ^2^Laboratoire de Mécanique et d'Acoustique, Centre National de la Recherche Scientifique, UPR 7051, Equipe Sons, Aix-Marseille Université, Centrale MarseilleMarseille, France; ^3^Laboratoire d'Accidentologie, de Biomécanique et d'Étude du Comportement HumainNanterre, France

**Keywords:** sound localization, perceptual learning, procedural learning, head-related transfer function, individual differences

Reason for Corrigendum:

Due to an oversight, there was a mistake in the Figure 2 as published. The black plots in the published Figure 2 were accidentally replaced with a duplicate of Figure 8. The correct version of Figure 2 appears below, this figure corresponds with the written data in the main article text.

**Figure 2:**
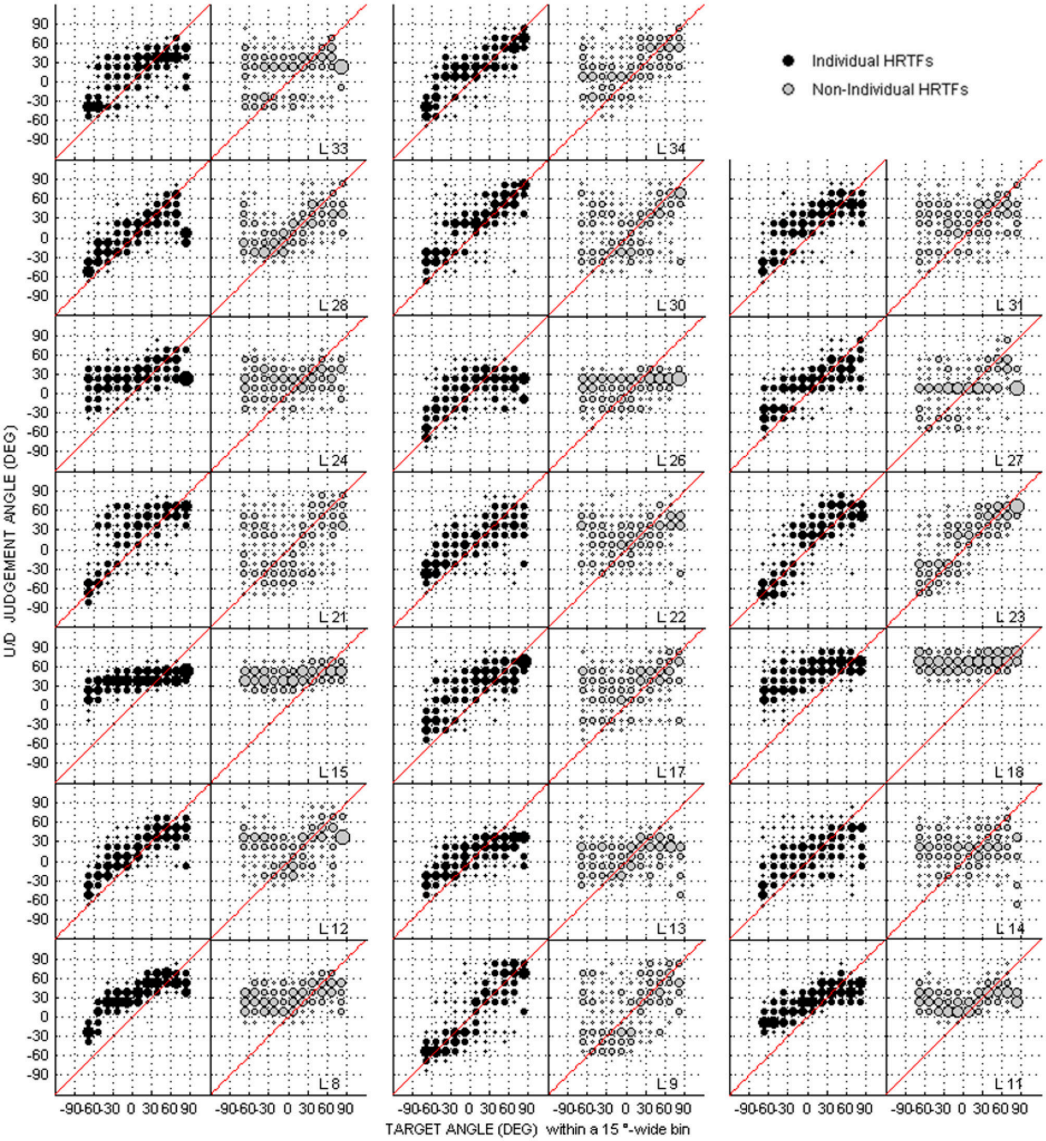
**Individual judgment position against target position with individual and non-individual HRTFs (black and gray dots, respectively) at the pre-test in the up/down dimension**. Each panel couple is for a different listener (*N* = 20).

The authors apologize for the mistake.

This error does not change the scientific conclusions of the article in any way.

## Author contributions

GA wrote the corrigendum. SS and AG viewed and approved this Corrigendum.

### Conflict of interest statement

The authors declare that the research was conducted in the absence of any commercial or financial relationships that could be construed as a potential conflict of interest.

